# Clinical and genetic attributes of congenital anomalies ascertained at a tertiary care hospital in Rawalpindi, Pakistan

**DOI:** 10.12669/pjms.39.6.7408

**Published:** 2023

**Authors:** Fatima Shaheen, Qaisar Shehzad Humayoon, Sajid Malik, Sara Mumtaz

**Affiliations:** 1 Fatima Shaheen, M.Phil, Human Genetics Program, Department of Zoology, Quaid-i-Azam University, Islamabad, Pakistan; 2 Qaisar Shehzad Humayoon, MBBS, FCPS Department of Pediatrics, Holy Family Hospital, Rawalpindi Medical University, Rawalpindi, Pakistan; 3 Sajid Malik, PhD, Human Genetics Program, Department of Zoology, Quaid-i-Azam University, Islamabad, Pakistan; 4 Sara Mumtaz, PhD Human Genetics Section, National University of Medical Sciences, Rawalpindi, Pakistan

**Keywords:** Birth defects, Consanguineous marriages, Neurological disorders, Prevalence pattern, Descriptive epidemiology

## Abstract

**Objective:**

Congenital anomalies (CA) or birth defects cause substantial healthcare burden in developing countries. There are few studies from Pakistan on the prevalence-pattern of CA. The purpose of this study was to determine the prevalence-pattern and genetic attributes of CA at a tertiary care facility in Rawalpindi, Pakistan.

**Methods:**

In a cross-sectional study design, patients with CA were ascertained from Pediatric and Neonatal Section of Holy Family Hospital, Rawalpindi from March-2022 to June-2022. International Classification of Diseases (ICD-10) and Online Mendelian Inheritance in Man (OMIM) databases were utilized for uniformity in classification. The pattern of CA as well as familial/sporadic nature, syndromic/isolated presentations, and prenatal consanguinity were estimated. Descriptive summaries were generated.

**Results:**

A total of 517 independent cases with certain types of CA were recruited. There were eight major and 70 minor categories. Among the major categories, neurological disorders (39.1%) were predominating followed by neuromuscular disorders (21.1%), limb defects (13.5%), musculoskeletal defects (7.4%), blood disorders (4.3%), orofacial defects (3.9%), metabolic disorders (3.7%), and Others (7.1%). The sporadic cases were in majority (72.5%) compared to familial cases (27.5%). Further, 63% patients had syndromic presentations and there were 37% cases with isolated appearances. A total of 70% cases had parental consanguinity.

**Conclusion:**

The majority of anomalies were of preventable nature and certain healthcare measures including antinatal care and counseling can be adopted to minimize their burden. Additionally, there is an urgent need to raise awareness of the negative consequences of consanguineous marriages, which constitute a significant risk factor in cases with inherited CA.

## INTRODUCTION

Congenital anomalies (CA) or birth defects can be functional or structural anomalies that occur during fetal development and may be noticed before, at or after birth. CA are estimated to affect 3-6% of newborns worldwide and are associated with hundreds of thousands of deaths. However, as statistics frequently do not take aborted pregnancies and stillbirths into account, the actual number of cases may be significantly greater.^1^ Non-genetic risk factors for CA include maternal illnesses, nutritional deficiencies, infections, and environmental toxins.^2^,^3^ Meanwhile, socioeconomic disadvantage, disparities in health or misinformation may compromise prevention efforts.^4^ A significant percentage of CA are brought on by genetic anomalies, such as chromosomal abnormalities or disorders due to single gene defect.^5^

Congenital and inherited abnormalities are a significant health burden in Pakistan.^6^ The healthcare system is unable to handle and treat people who are affected by these abnormalities. Thus, such abnormalities have a profound impact on quality of life, psychology and economy of people involved, their families and the society in general. CA are responsible about 6-9% of perinatal mortalities in Pakistan.^7^ Here, a number of factors including insufficient maternal malnutrition, prenatal care, a poor socioeconomic environment, rural origin, and high rate of consanguinity, have been linked to the high incidence of CA.^8^-^10^

Literature search revealed few of the hospitals-based studies that were conducted to assess CA prevalent in Pakistani population. To ascertain the prevalence and pattern of CA, one such study was carried out in the neonatal unit of the Combined Military Hospital, Kharian, Pakistan. Neonatal CA cases accounted for about 7% of the total admissions. Among the several body systems that were afflicted, the central nervous system (CNS) in 20% cases, followed by the musculoskeletal system in 19%, genitourinary system in 15%, cardiovascular system in 13%, ear, eye, face, and neck in 12%, digestive system in 8%, and skin in 6%. The authors remarked that CNS were the most frequently affected system and CA were not uncommon in our society.^11^ Another descriptive cross-sectional study was conducted at Mardan Medical Complex, Mardan, and infants with CA born between May 2016-April 2017 were enrolled. A total of 1.23% of newborns exhibited CA. The most frequent anomalies were hydrocephalus that occurred in 27% of cases, followed by anencephaly in 18%, meningomyelocele in 11%, and encephalocele that prevailed in 9% of cases. In 62% of patients, parental consanguinity was confirmed, while 71% of cases lacked maternal folic acid intake.^12^

Furthermore, a cross-sectional study carried out at the Department of Pediatrics, Civil Hospital Karachi, revealed that at least one birth abnormality was evident in 4% of the pediatric cases who visited the hospital. The most frequent of these were urogenital system (20%), eye (17%), musculoskeletal system (13%), oral cavity (12%), body wall defects (12%), and CNS (11%). It was concluded that socioeconomic, maternal, dietary, and educational variables were linked to the occurrence of CA.^13^

In order to better understand the prevalence-pattern of CA in a multi-ethnic Pakistani population, this study was carried out at Holy Family Hospital which is a tertiary care hospital in Rawalpindi, receiving a large influx of patients from twin-cities Rawalpindi-Islamabad. This study further elaborates the clinical and genetic attributes of CA which would be helpful in the understanding the heterogeneity among these disorders.

## METHODS

In a cross-sectional study design, the patients with CA were recruited from the in-patient department of the Pediatric and Neonatal Section of Holy Family Hospital, Rawalpindi. As majority of the participants were under the legal age to give consent or were unable to give consent due to a disability, informed consent was obtained from the patient’s parents or guardians. Subjects with anomalies of traumatic nature or infections were not included. The study was conducted from March-2022 to June-2022. The study was approved by the Ethical Review Committees of Quaid-i-Azam University (DAS-19; July 3, 2019).

### Classification of anomalies

The malformations were diagnosed by team of specialized doctors and neonatologists. Further, the description of CA was taken from standard coding system of the International Classification of Diseases (ICD-10) and Online Mendelian Inheritance in Man (OMIM) databases. The CA were divided into major categories according to the involvement of respective organ-systems. Major classification of anomalies was as follows: neurological disorders, neuromuscular disorders, musculoskeletal defects, limb defects, blood disorders, orofacial defects, and metabolic disorders. If multiple anomalies were present, the primary major anomaly was taken into account (i.e., neurological, neuromuscular or cardiac), and the additional symptoms were listed as associations (i.e., syndromic).

The index cases were further classified on the basis of isolated/syndromic and familial/sporadic presentations. A pedigree consisting of three generations was constructed for each case but only the index case from a particular family was included in the analyses. Descriptive statistics were used to determine the significance of the distribution, and Chi-square and Fisher-exact tests were applied. For the CA, proportions and corresponding 95% confidence intervals (CI) were calculated.

## RESULTS

This study included 517 independent cases (291 males, 226 females) with certain type of CA. Patients up to five years comprised 72% of the sample, majority originated from Punjab province (78%) and urban areas (56%) (Table-I).

**Table-I:** Demographic attributes of patients.

Variables	Gender		Total	

	Male	Female	No.	%
***Age categories (years)***				
Up to 5	215	159	374	72.3
>5-9	24	23	47	9.1
>9	52	44	96	18.6
Total	291	226	517	100.0
	Chi2=0.915; P=0.633			
***Province***				
Punjab	217	188	405	78.3
Khyber Pakhtunkhwa	27	14	41	7.9
Azad Jammu Kashmir	20	15	35	6.8
Islamabad	27	9	36	7.0
	Chi2=7.87; P=0.049			
***Rural/urban origin***				
Rural	140	88	228	44.1
Urban	151	138	289	55.9
	Chi2=4.34; P=0.037			
***Mother tongue***				
Punjabi	145	132	277	53.6
Pashto	59	31	90	17.4
Urdu	19	22	41	7.9
Pahari	25	11	36	7.0
Hindko/Pothawari	22	19	41	7.9
Others	21	11	32	6.2
	Chi2=21.59; P=0.119			

### Pattern of congenital anomalies

The CA were classified into eight major categories: neurological disorders (39.1%), neuromuscular disorders (21.1%), limb defects (13.5%), musculoskeletal defects (7.4%), blood disorders (4.3%), orofacial defects (3.9%), metabolic disorders (3.7%), and Others (7.1%) (Table-II).

**Table-II:** Distribution of major categories of congenital anomalies with respect to gender and familial/sporadic nature

Major category	Index cases		Total		Proportion	95% CI	Familial/sporadic	
	
	Male	Female	No.	%			Sporadic	Familial
Neurological disorders	117	85	202	39.1	0.391	0.349-.433	155	47
Neuromuscular disorders	56	53	109	21.1	0.211	0.176-.246	71	38
Limb defects	43	27	70	13.5	0.135	0.106-.165	49	21
Musculoskeletal defects	20	18	38	7.4	0.074	0.051-.096	29	9
Blood disorders	11	11	22	4.3	0.043	0.025-.060	14	8
Orofacial defects	14	6	20	3.9	0.039	0.022-.055	16	4
Metabolic disorders	10	9	19	3.7	0.037	0.021-.053	18	1
Others	20	17	37	7.1	0.072	0.049-.094	23	14
Total	291	226	517	100.0	1.000	1.000-.000	375	142
	Chi2=4.31;P=0.74						Chi2=13.41; P=0.627	

There were at least 70 minor entities (Table-III). Among the neurological disorders, there was highest representation of hydrocephaly, spina bifida and Down syndrome (n=48, 48 and 35, respectively). The neuromuscular defects were represented by cerebral palsy types; congenital (n=79) and late-onset (post-asphaxial, post-meningitis, post-traumatic; n=30). Among the limb defects, there was highest representation of talipes (n=39) and polydactyly (n=15). Among the musculoskeletal defects, three most common types were muscular dystrophy (n=7), arthrogryposis (n=5) and osteopetrosis (n=5).

**Table-III:** Major and minor categories of congenital anomalies.

Major/minor category	Frequency	Proportion	95% CI	ICD-10^14^	OMIM^15^
Neurological disorders	202	0.391	0.349-0.433		
Hydrocephaly	48	0.093	0.068-0.118	G91.9	236600
Spina bifida	48	0.093	0.068-0.118	Q05	182940
Down syndrome	35	0.068	0.046-0.089	Q90	190685
Developmental delay	22	0.043	0.025-0.060	Z13.42	618330
Intellectual disability	19	0.037	0.021-0.053	F03	300243
Epilepsy	11	0.021	0.009-0.034	G40	117100
Microcephaly	11	0.021	0.009-0.034	Q02	251200
Encephalocele	5	0.010	0.001-0.018	Q01.9	607132
Edwards syndrome	2	0.004	-0.001-0.009	Q91.3	601161
Leukodystrophy	1	0.002	-0.002-0.006		607694
Neuromuscular disorders	109	0.211	0.176-0.246		
Cerebral palsy (congenital)	79	0.153	0.122-0.184	G80	605388
Cerebral palsy (late-onset)	30	0.058	0.038-0.078		
Limb defects	70	0.135	0.106-0.165		
Talipes (all types)	39	0.075	0.053-0.098	Q66.0	119800
Polydactyly (all types)	15	0.029	0.015-0.043	Q69.9	174200, 174400
Amputation (transverse)	5	0.010	0.001-0.018	Q73.0, Q72.0	217100
Brachydactyly (all types)	4	0.008	0.000-0.015	Q68.81	113000
Radial hemimelia	2	0.004	-0.001-0.009	Q73.8	
Syndactyly (all types)	2	0.004	-0.001-0.009	Q70	609815
Club hand	1	0.002	-0.002-0.006	Q71.4	
Fibular hemimelia	1	0.002	-0.002-0.006		
Split hand	1	0.002	-0.002-0.006	Q72.7	183600
Musculoskeletal defects	38	0.074	0.051-0.096		
Muscular Dystrophy	7	0.014	0.004-0.024	G71.0	310200
Arthrogryposis	5	0.010	0.001-0.018	Q74.3	108120
Osteopetrosis	5	0.010	0.001-0.018	Q78.2	259710
Dwarfism, skeletal dysplasia	4	0.008	0.000-0.015	E34.3	100800
Developmental dysplasia of hip	3	0.006	-0.001-0.012	Q65.8	142700
Multiple exostosis	3	0.006	-0.001-0.012	Q78.6	133700
Limb hypotonia	2	0.004	-0.001-0.009	P94.2	300868
Osteogenesis imperfecta	2	0.004	-0.001-0.009	Q78.0	166200
Achondroplasia	1	0.002	-0.002-0.006	Q77.4	100800
Apert syndrome	1	0.002	-0.002-0.006	Q87.0	101200
Crouzon syndrome	1	0.002	-0.002-0.006	Q75.1	123500
Dystrophic dwarfism	1	0.002	-0.002-0.006	E34.5	100800
Pfeiffer syndrome	1	0.002	-0.002-0.006	B27.0	101600
Rickets	1	0.002	-0.002-0.006	E34.4	100800
Scoliosis	1	0.002	-0.002-0.006	M41	181800
Blood disorders	22	0.043	0.025-0.060		
Thalassemia	12	0.023	0.010-0.036	D56	613985
Anemia	4	0.008	0.000-0.015	D64.9	
Hemophilia	2	0.004	-0.001-0.009	D66	306700
Pancytopenia	2	0.004	-0.001-0.009	D61.0	
Fanconi anemia	1	0.002	-0.002-0.006	D61.09	227650
Sickle cell anemia	1	0.002	-0.002-0.006	D57.1	603903
Orofacial	20	0.039	0.022-0.055		
Cleft lip and palate	8	0.015	0.005-0.026	Q37	119530
Cleft palate only	7	0.014	0.004-0.024	Q35	119540
Dysmorphic face	2	0.004	-0.001-0.009		
Choanal atresia	1	0.002	-0.002-0.006	Q30.0	608911
Cleft lip only	1	0.002	-0.002-0.006	Q36	600625
Pierre-Robin syndrome	1	0.002	-0.002-0.006	Q87.0	261800
Metabolic disorder	19	0.037	0.021-0.053		
Storage disorders	7	0.014	0.004-0.024		
Cystic fibrosis	4	0.008	0.000-0.015	E84.0	219700
Gaucher disease	3	0.006	-0.001-0.012	E75.2	230800
Hurler syndrome	2	0.004	-0.001-0.009	E76.0	607014
Wilson disease	1	0.002	-0.002-0.006	E83.0	277900
Mucopolysaccharidosis	1	0.002	-0.002-0.006	E76.3	252800
Niemann-Pick disease	1	0.002	-0.002-0.006	E75.2	257200
Others	37	0.072	0.049-0.094		
Congenital heart defects	11	0.021	0.009-0.034	Q23.4	614954
Deaf and Mute	6	0.012	0.002-0.021	H91.3	304500
Ichthyosis	6	0.012	0.002-0.021	L85.0	242300
Omphalocele	3	0.006	-0.001-0.012	Q79.2	164750
Albinism	1	0.002	-0.002-0.006	E70.3	203100
Ambiguous genitalia	1	0.002	-0.002-0.006	Q56.4	250790
Ectodermal dysplasia	1	0.002	-0.002-0.006	Q82.4	305100
Immunodeficiency	1	0.002	-0.002-0.006	D89.9	
Inflammatory bowel disease	1	0.002	-0.002-0.006	K50-52	612567
Keratoderma	1	0.002	-0.002-0.006	L40.3	144200
Pulmonary hypertension	1	0.002	-0.002-0.006	I27.0	178600
Retinitis pigmentosa	1	0.002	-0.002-0.006	H35.5	603937
Retinoblastoma	1	0.002	-0.002-0.006	C69.2	180200
Speech impairment	1	0.002	-0.002-0.006	R47.0	
Systemic lupus erythematosus	1	0.002	-0.002-0.006	M32	152700

### Familial/sporadic nature, parental consanguinity, isolated/syndromic presentations

The sporadic cases were in majority (n=375, 72.5%) compared to familial (n=142, 27.5%) (Table-II). There were 756 (433 males, 332 females; P=0.0104) (Table-IV) affected subjects among all families. A total of 70% of cases had parental consanguinity. The highest consanguinity was observed in musculoskeletal defects and metabolic disorders (89%) followed by orofacial defects (85%), while lowest rate of consanguinity was witnessed in neuromuscular disorders (63%). The differences in the rate of consanguinity among the major categories were statistically highly significant (Table-IV).

**Table-IV:** Parental consanguinity and isolated/syndromic nature of major categories of congenital anomalies.

Major category	Parental consanguinity		Total index cases	Isolated/syndromic nature		Total affected in all families		

	Yes	No		Isolated	Syndromic	Male	Female	Total
Neurological disorders	132	70	202	45	157	156	119	275
Neuromuscular disorders	69	40	109	1	108	83	83	166
Limb defects	50	20	70	59	11	66	42	108
Musculoskeletal defects	34	4	38	19	19	39	24	63
Blood disorders	14	8	22	19	3	19	17	36
Orofacial defects	17	3	20	14	6	17	7	24
Metabolic disorders	17	2	19	7	12	11	11	22
Others	28	9	37	27	10	42	29	71
Total	361	156	517	191	326	433	332	765
	Chi2=17.8; P=0.013			Chi2= 202.6; P<0.0001		Chi2=7.33; P=0.359		

Further, at least 63% of cases had syndromic presentations and there were 37% of cases with isolated appearances (Table-IV). Syndromic presentations were common among neuromuscular and neurological disorders (99% and 78%, respectively), whereas isolated presentations were conspicuous in blood disorders, limb defects and metabolic defects (86%, 84% and 70%, respectively) (P<0.0001). Among the syndromic cases, most common associated defects were developmental delay (156), sensorineural/pinna defects (78), and epilepsy (51) (data not shown).

## DISCUSSION

This hospital-based study was conducted to assess the prevalence-pattern and phenotypic manifestations of CA in the multiethnic population of Rawalpindi/Islamabad. The most commonly observed category of CA was neurological disorders followed by neuromuscular disorders, limb defects, and musculoskeletal anomalies. The results are comparable to other studies conducted in Pakistan and other countries which showed highest prevalence of CNS defects.^11^,^12^ In this study, the most frequent neurological anomalies were hydrocephaly and spina bifida followed by Down syndrome, while most frequent neuromuscular disorder was cerebral palsy.

The reason of the high prevalence of neural tube defects, i.e., spina bifida and associated hydrocephaly could be a lack of maternal folic acid use during the period of conception and early development.^16^ Down syndrome is caused by trisomy of chromosome 21 and associated with the advance age of mother.^17^ There is no screening test available in the local hospital. It can be detected between weeks 10 and 14 of pregnancy with a combination of ultrasound scan and blood test. The most frequent neuromuscular disorder was cerebral palsy. Cerebral palsy could occur due to different reason such as preterm delivery, intrauterine infection and placental abnormalities, fetal growth restriction and birth asphyxia.^18^ In a recent study carried out at a tertiary care hospital of Karachi, the CNS anomalies were dominating with 22% hydrocephalus cases and 20% anencephaly cases. However, renal abnormalities and gastrointestinal anomalies were the next most typical birth malformations.^19^ While in our data the after neurological and neuromuscular anomalies, limb defects and musculoskeletal disorders were more prevalent.

These findings are consistent with another study carried out in the tertiary care hospital of Mardan, Pakistan in which CNS cases were prevalent and were succeeded by musculoskeletal anomalies. Our study and previous studies in Pakistan showed high prevalence of neurological disorders in children. The burden of neurological diseases remains high in developing countries. This could be due to high incidence of prematurity, asphyxia, neonatal infectious, consanguinity and infections of CNS.^20^

Another finding in our study is the relatively high prevalence of limb defects that was not reported separately in previous hospital-based studies from Pakistan. Due to their minor nature, fingers/toes defects like syndactyly, polydactyly, brachydactyly and camptodactyly, may be overlooked in clinical practice.^21^ On the other hand, epidemiological studies carried out in the general populations of Pakistan, i.e., Sialkot, Hazara and Kurram Agency, showed high prevalence of limb defects.^10^,^22^,^23^ Talipes and polydactyly were predominating limb defects in our study. Talipes, if not treated early can cause disability, while polydactyly is more like a cosmetic problem.

In the present sample, sporadic cases were more common than familial cases (72.5% vs. 27.5%). This finding is consistent with a recent epidemiological study conducted in Sialkot and Hazara populations, Pakistan.^10^,^22^ Sporadic anomalies could arise mainly due to nongenetic factors like environmental conditions, maternal factors, infection and exposure to chemicals and drugs during pregnancy, and physical factors such as ionizing radiation and hyperthermia.^24^ However, we cannot exclude the possibility that a sizable number of sporadic cases have a genetic cause and/or their familial nature could not be determined due to limitations in data collection.

In the current study, parental consanguinity was observed in 70% of the cases. Among the major categories, the highest consanguinity was observed in musculoskeletal defects and metabolic disorders (89%) followed by orofacial defects (85%), which is much higher than the background consanguinity in the Pakistani population and may indicate high contribution of recessive genes.^6^,^25^,^26^ This study represents a detailed picture of CA prevalent in local in the population of Rawalpindi. To the best of our knowledge, this is the first study reporting such a large cohort of CA. From community health perspective, this study is a part of public health surveillance of major congenital anomalies which are cause of disabilities. Our scientific findings highlight group of disorders which have severe impact on the socio-psychological well-being of patients and families and require long-term management. These data would be very useful for policy making, awareness compaigns, community management and resource allocation. Based on this study further large-scale studies can be designed for this population to get a clear estimation of types and patterns of CA. In a prospective study, it would be worthwhile to identify extended and multi-generational families for molecular genetic study which would be useful for genetic counsling.

### Limitations

This study is based on data from a single hospital and may not be representive of other communities/areas. In order to make policies, we need a multicenter study encompassing other hospitals of the city.

## CONCLUSION

The majority of the CA witnessed in this sample are of preventable nature and their burden can be significantly reduced by undertaking certain precautions and healthcare measures. To reduce the fraction of CA that are preventable, healthcare administrators must place a strong emphasis on primary prevention through genetic counseling, prenatal care, immunization, nutrition, and medications. There is also a dire need to spread awareness about the detrimental effects of consanguinity which is a major risk factor in the case of inherited CA.

### Authors’ Contribution:

**SM_K_** & **SM_z_:** Conceived, designed and supervised the study. **SM_K_:** Statistical analysis. **FS** and **QSH:** Data collection and manuscript writing. **SMK** & **SMZ:** Responsible and accountable for the accuracy and integrity of data.
